# Sulfate transporters in the plant’s response to drought and salinity: regulation and possible functions

**DOI:** 10.3389/fpls.2014.00580

**Published:** 2014-10-29

**Authors:** Karine Gallardo, Pierre-Emmanuel Courty, Christine Le Signor, Daniel Wipf, Vanessa Vernoud

**Affiliations:** ^1^Institut National de la Recherche Agronomique, UMR1347 Agroécologie, DijonFrance; ^2^Zurich-Basel Plant Science Center, Department of Environmental Sciences, Botany, University of Basel, BaselSwitzerland; ^3^Université de Bourgogne, UMR1347 Agroécologie, DijonFrance

**Keywords:** sulfate, transporters, abiotic stresses, *M. truncatula*, *Arabidopsis*

## Abstract

Drought and salinity are two frequently combined abiotic stresses that affect plant growth, development, and crop productivity. Sulfate, and molecules derived from this anion such as glutathione, play important roles in the intrinsic responses of plants to such abiotic stresses. Therefore, understanding how plants facing environmental constraints re-equilibrate the flux of sulfate between and within different tissues might uncover perspectives for improving tolerance against abiotic stresses. In this review, we took advantage of genomics and post-genomics resources available in *Arabidopsis thaliana* and in the model legume species *Medicago truncatula* to highlight and compare the regulation of sulfate transporter genes under drought and salt stress. We also discuss their possible function in the plant’s response and adaptation to abiotic stresses and present prospects about the potential benefits of mycorrhizal associations, which by facilitating sulfate uptake may assist plants to cope with abiotic stresses. Several transporters are highlighted in this review that appear promising targets for improving sulfate transport capacities of crops under fluctuating environmental conditions.

## INTRODUCTION

Drought, the incidence of which is expected to increase with climatic changes, is one of the major abiotic constraints on agricultural productivity. Because drought is often associated with salinity, one challenge for sustainable agriculture is to breed crops for enhanced tolerance to both stresses. This requires an understanding of the adaptive mechanisms allowing plants to survive in low-water and high-salt environments. Sulfur is a key component in helping plants to cope with such abiotic stresses (for review, see Chan et al., 2013). For example, sulfur is used for the synthesis of glutathione, which acts in the maintenance of the cellular redox balance and mitigates damage caused by reactive oxygen species. Most of the sulfur taken up by plants is in the form of sulfate, and several studies point to a role of this anion in the plant response to drought and salinity in relation to the phytohormone abscisic acid (ABA), a major regulator of leaf stomatal conductance (Wilkinson and Davies, 2002). It was proposed that sulfate acts as a primary signal to enhance the anti-transpirant effect of ABA reaching the stomata in leaves (Ernst et al., 2010). More recently, Cao et al. (2014) provided evidence for a significant co-regulation of sulfur and ABA metabolisms in *Arabidopsis* that may help to combat environmental stresses. Such metabolic adjustments undoubtedly rely on the plant’s ability to absorb and distribute sulfate to the different organs in amounts sufficient to fulfill requirements.

Major advances have been made toward identifying and characterizing the transporters involved in the uptake, distribution, or eﬄux of sulfate from the vacuoles, especially in *Arabidopsis* (Buchner et al., 2004 and references therein). The investigation of the contribution of sulfate transporters (SULTR) to abiotic stress tolerance has begun more recently. Cao et al. (2014) proposed a role for SULTR3;1 in helping plants to cope with environmental stresses by providing sulfate for the synthesis of cysteine that serves as a sulfur donor during ABA biosynthesis. With the advances made over the last decade in the integration of “omics” data, gene expression atlases are now available for several species, giving access to the regulation of any gene of interest in different conditions. In this review, we took advantage of these resources to highlight the regulation of *SULTR* genes in response to drought and salinity. We focus on *Arabidopsis* and *M. truncatula*, the latter being a wild legume species originating from the Mediterranean basin that makes use of symbiotic associations to obtain nutrients and that has evolved to develop a tolerance to extreme environmental conditions including drought and salinity (Friesen et al., 2010). After a search of the *SULTR* sequences in *M. truncatula* and of their closest homologs in *Arabidopsis*, we discuss and compare their regulation and possible contribution to protection against unfavorable environmental conditions. We also highlight the potential benefit of using arbuscular mycorrhizal (AM) fungi to improve sulfate uptake.

## COMPARATIVE ANALYSIS OF *SULTR* GENE FAMILIES BETWEEN *Arabidopsis* AND *M. truncatula*

**FIGURE 1:**
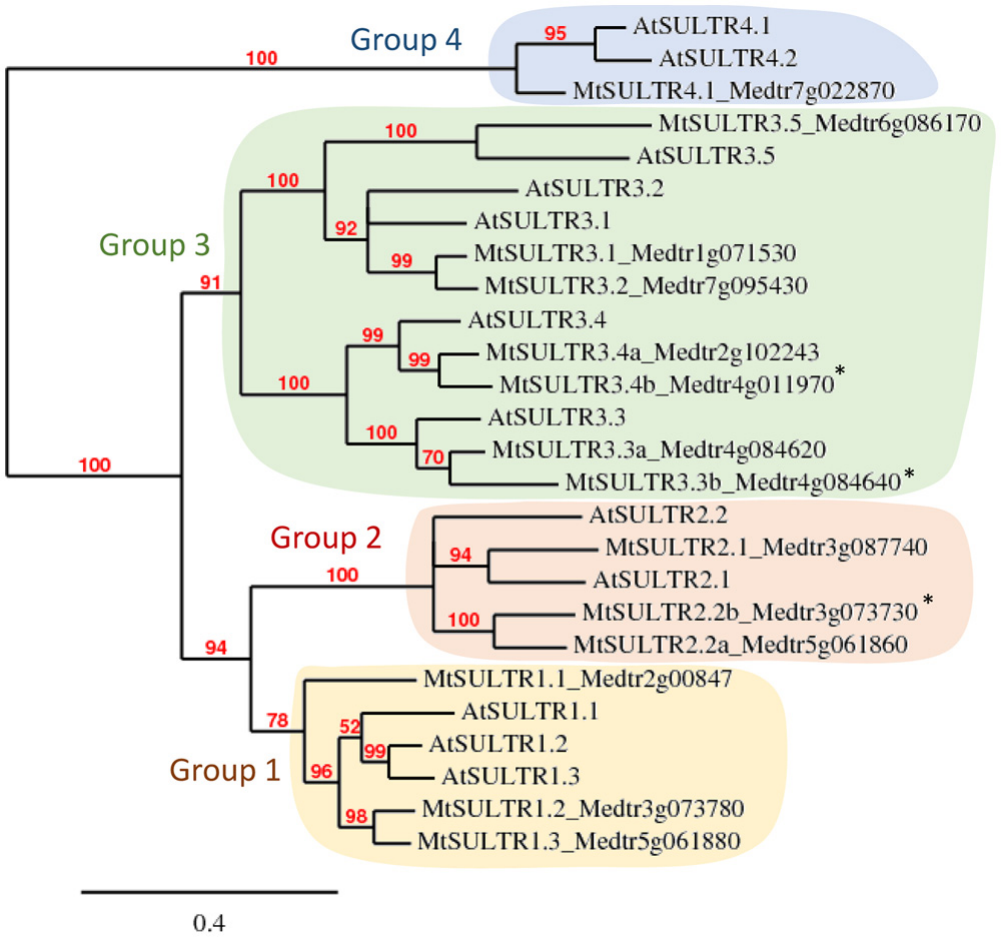
**Phylogenetic tree of *Medicago truncatula* and *Arabidopsis* sulfate transporters.** The Maximum likelihood phylogenetic tree was generated using all SULTR amino acid sequences available in the *Arabidopsis* and *M. truncatula* (Mt4.0v1) genomic resources. *, sequences for which there was no corresponding probe in the *Medicago* Gene Expression Atlas.

*Medicago truncatula* is an annual forage species adopted in 2001 as a model for legumes because of its small genome, compared to crop legumes such as pea, and its ability to perform symbiotic interactions with nitrogen-fixing rhizobia and AM fungi, like most legume species (Frugoli and Harris, 2001). The close relationship of the *M. truncatula* genome with that of pea (*Pisum sativum* L.) facilitates the transfer of information to the crop, and molecular markers have been developed for translational genomics between the two species (Bordat et al., 2011). *M. truncatula* is native to the arid and semi-arid environments of the Mediterranean. It is thus adapted to this climate, making it a good model to identify adaptation processes to low-water or high-salt stresses. Genomic resources were developed for this species that we used here to retrieve *SULTR* genes (*MtSULTR*). Fourteen genes homologous to the *Arabidopsis SULTR* genes (*AtSULTR*) were identified in the last *Medicago* genome version 4.0v1^1^. Phylogenetic analysis using SULTR full length amino-acid sequences allowed us to re-annotate the MtSULTRs and to refine their phylogenetic relationship with AtSULTRs (**Figure 1**). The corresponding neighbor-joining tree divided into four clusters matching the four groups described in *Arabidopsis* (Buchner et al., 2004), as previously observed by Casieri et al. (2013). Three MtSULTRs clustered with the three *Arabidopsis* transporters of high-affinity belonging to group 1, involved in sulfate uptake (SULTR1;1 and 1;2, Yoshimoto et al., 2007; Barberon et al., 2008) or in its distribution to sink organs (SULTR1;3, Yoshimoto et al., 2003). Three others MtSULTRs clustered with the two *Arabidopsis* members of group 2 that deliver sulfate to aerial parts and developing tissues (Takahashi et al., 2000; Awazuhara et al., 2005). Group 3 is the largest group, with seven members in *M. truncatula* compared to five in *Arabidopsis*. They play multiple roles, such as facilitating sulfate transport to aerial parts or controlling cysteine level in seeds and seedlings in tight interaction with ABA metabolism (Kataoka et al., 2004a; Zuber et al., 2010; Cao et al., 2014). One member of this group, SULTR3;1, is responsible for sulfate transport into chloroplasts (Cao et al., 2013). Within group 4, unlike *Arabidopsis* which contains two *SULTR4* genes, there was only one *M. truncatula* gene. It encodes a protein with high homology to AtSULTR4;1 which plays a major role in the eﬄux of sulfate from the vacuole lumen to the cytosol (Kataoka et al., 2004b). This suggests a unique function for MtSULTR4;1 in remobilizing the stored sulfate. This may apply to other species as there is only one transporter of group 4 with high homology to AtSULTR4;1 in pea (RNAseq data, Burstin J, personal communication) and rice (Kumar et al., 2011).

The recent transcriptome analysis of *M. truncatula* subjected to progressive drought (Zhang et al., 2014a) allowed us to investigate the transcriptional regulation of the *MtSULTR* gene family in response to this abiotic stress and in comparison with a salt stress response (Li et al., 2009). Data were downloaded from the Gene Expression Atlas (MtGEA)^2^, and expression fold-change between treated and non-treated samples was calculated (cutoff of 2.0, **Table 1**). Expression of three of the 14 *MtSULTR* genes (*MtSULTR2;2b*, *MtSULTR3;3b,* and *MtSULTR3;4b*, **Figure 1**) could not be investigated as there was no corresponding probe set in the Affymetrix chip used to build the MtGEA. To compare *SULTR* gene regulation between *M. truncatula* and *Arabidopsis*, we used transcriptomic data available in *Arabidopsis* for drought and salt stress experiments (Kilian et al., 2007; Huang et al., 2008; Perera et al., 2008; Nishiyama et al., 2012; Geng et al., 2013; Pandey et al., 2013; Wang et al., 2013; Ha et al., 2014). The studies showing the most substantial regulation of *SULTR* genes are included in **Table 1**. Results are discussed in the light of functional data available, mainly in *Arabidopsis*.

**Table 1:** Regulation of *SULTR* gene expression in *Medicago truncatula* and *Arabidopsis* subjected to drought and salt stress.

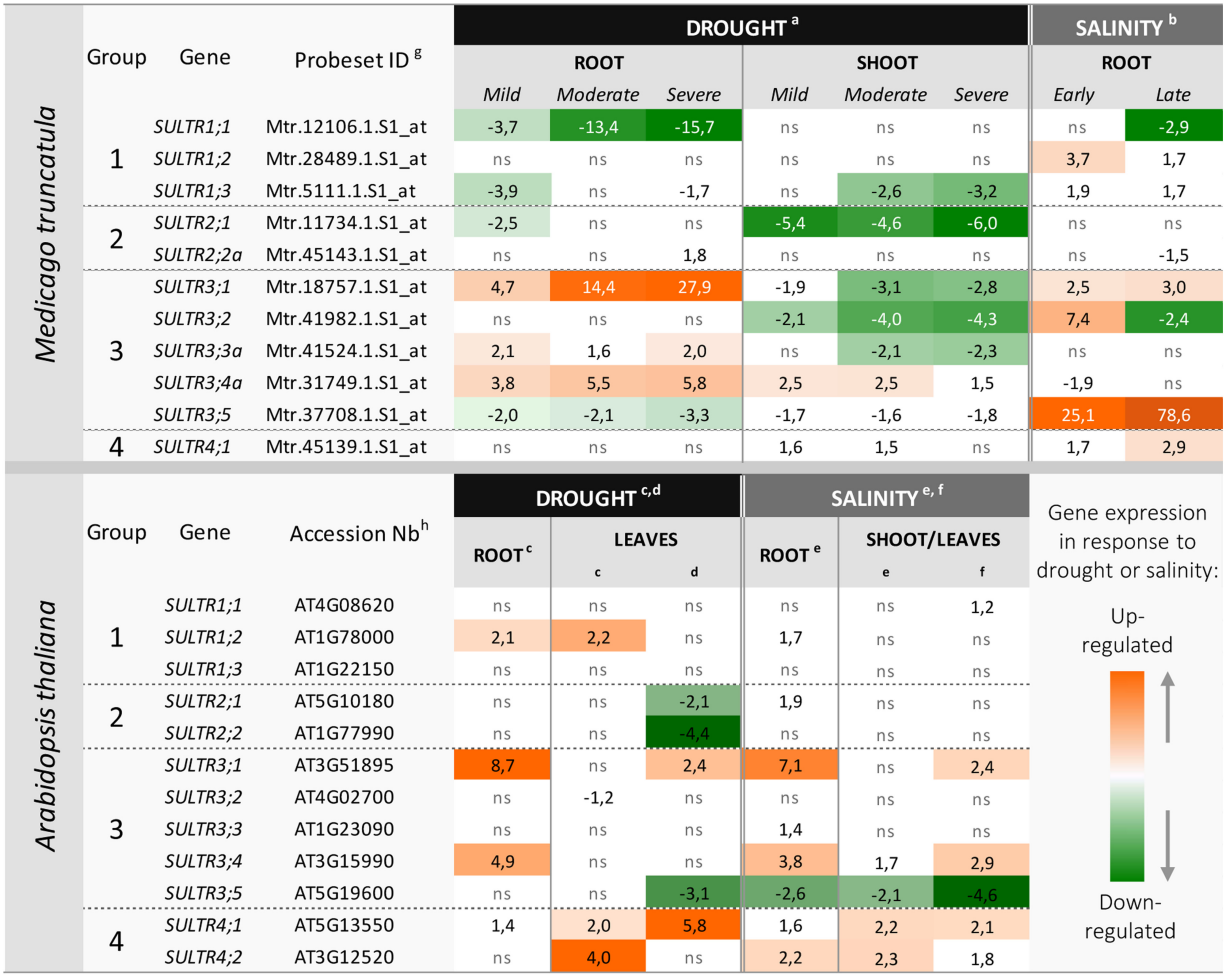

*SULTR gene regulation from: (a) Zhang et al. (2014a): mild, moderate or severe water stress (corresponding to 7, 10, or 14 days of water withdrawal, respectively) applied on 24 day-old M. truncatula plants. (b) Li et al. (2009): young seedlings (2 days) treated with 180 mM of NaCl for 6 h (early response) or 2 days (late reponse); (c) Ha et al. (2014): aerial portions of 24 day-old plants detached and exposed to dehydration on paper towels for 4 h; (d) Pandey et al. (2013): 3 week-old plantlets grown for 9 days on soil with a moisture level below 30%; (e) Kilian et al. (2007): 150 mM NaCl applied to Arabidopsis seedlings in vitro; (f) Wang et al. (2013): 10 day-old seedlings grown for 4 days on a medium supplemented with 100 mM NaCl. For each MtSULTR gene, data for the correponding probeset ID (g) were downloaded from the Medicago Gene Expression Atlas at http://mtgea.noble.org/v3/experiments. (h) Genbank accession number of the Arabidopis SULTR genes.The values refer to gene expression fold change between treated and non-treated samples. Changes in gene expression of at least twofold are highlighted using a color scale; ns, non-significant change in gene expression in response to drought or salt stress.*

## *SULTR* OF GROUP 3 ARE STRONGLY REGULATED BY ABIOTIC STRESSES IN ROOTS

Of particular interest is the up-regulation of the *SULTR3;1* gene in roots of both species subjected to drought and salt stress. Interestingly, the expression of *AtSULTR3;1* is enhanced by ABA and required for cysteine synthesis (Cao et al., 2014). Cysteine, whose precursor is sulfate, plays a key role in ABA synthesis as it serves as sulfur donor for the sulfuration of molybdenum, a co-factor needed in its sulfurylated form for the last reaction in the pathway (Xiong et al., 2001). The cysteine formed may also serve for the synthesis of the stress-defense compound glutathione. Cao et al. (2014) proposed that sulfur metabolism and ABA biosynthesis interplay to ensure sufficient cysteine for ABA production under abiotic stresses. From these data and the reported plastid-localization of AtSULTR3;1 (Cao et al., 2013), it is tempting to speculate on a role for this transporter in directing the flux of sulfate toward cysteine biosynthesis in the root plastids that may further be used for ABA production in response to both abiotic stresses. In *M. truncatula*, SULTR3;1 has not been functionally characterized. However, the gene is up-regulated in response to both abiotic stresses (**Table 1**) and co-localizes with quantitative trait loci (QTL) regions for salt tolerance (Friesen et al., 2010; Arraouadi et al., 2012), as also observed for *AtSULTR3;1* (El-Soda et al., 2014; Zhang et al., 2014c). This makes *MtSULTR3;1* a potential target for modulating the abiotic stress response in legumes. In addition, *MtSULTR3;1* expression is higher at late stages of water stress, i.e., severe water stress in **Table 1**, known to be associated with ABA biosynthesis in roots (Goodger and Schachtman, 2010), suggesting that MtSULTR3;1 could be closely linked in its action with ABA production, as is the case in *Arabidopsis* (Cao et al., 2014). Another gene of group 3 (*AtSULTR3;4, MtSULTR3;4a*) is co-expressed in roots with *SULTR3;1* in response to drought in the two species and in response to salt stress in *Arabidopsis* (**Table 1**). The reduced ABA content in seedlings for the two mutants *Atsultr3;1* and *Atsultr3;4* suggests a role for both genes in relation to ABA production. The subcellular localization of SULTR3;4 is unknown. Investigating spatial and subcellular localizations in roots for both transporters might help to decipher whether they can have a coordinated function or a functional redundancy in this tissue. It should be noted that in contrast to *Arabidopsis*, *MtSULTR3;1* and *MtSULTR3;4a* are differentially regulated in response to salt stress (only *MtSULTR3;1* is up-regulated) and that a second *MtSULTR3;4* gene (*MtSULTR3;4b*, **Figure 1**) exists whose response to salt stress is currently unknown.

In *M. truncatula*, the expression of another group 3 *SULTR* (*MtSULTR3;5*) is strongly up-regulated in roots subjected to salt stress (up to 78-fold; **Table 1**). Its closest *Arabidopsis* homolog, *AtSULTR3;5*, shows opposite trends of expression in roots with a consistent down-regulation in response to salinity. This suggests distinct roles or transcriptional regulation of *SULTR3;5* between the two species. In the legume species *Lotus japonicus*, the *SULTR3;5* homolog *SST1 (Symbiotic Sulfate Transporter 1)* is necessary for nodule formation and essential for the symbiotic supply of sulfur to the bacteria (Krusell et al., 2005). In this connection, Varin et al. (2010) identified sulfur supply as necessary for proper accumulation of nitrogenase and leghaemoglobin, two proteins rich in sulfur amino acids and needed for nitrogen fixation. This highlights the importance of maintaining efficient sulfate transport systems in nodules to exploit the nitrogen-fixing capacity of legume plants in agroecological systems. *MtSULTR3;5* is strongly expressed in nodules (Roux et al., 2014) and studies are ongoing to understand the function of MtSULTR3;5 in nodules and to deciphering its contribution to the salt stress response.

## RE-EQUILIBRATION OF SULFATE FLUX IN AERIAL PARTS IN RESPONSE TO ABIOTIC STRESSES

In contrast to the functional SST1 (Krusell et al., 2005), AtSULTR3;5 is a non-functional transporter by itself (Kataoka et al., 2004a). This transporter forms a complex with AtSULTR2;1, thus enhancing its sulfate import activity into cells of root vascular tissues for loading into the xylem and transfer to aerial parts, especially when sulfur availability is limited (Takahashi et al., 2000; Kataoka et al., 2004a). The flux of sulfur from roots to shoots is in part controlled by microRNA(Mir)395, which limits expression of *SULTR2;1* to xylem parenchyma, thus enhancing sulfate translocation to aerial parts (Kawashima et al., 2011). Interestingly, Mir395 is up-regulated in response to drought stress in rice (Zhou et al., 2010) and under high salinity conditions in maize (*Zea mays* L.; Ding et al., 2009), suggesting it participates in abiotic stress responses, presumably by maintaining the flux of sulfur toward aerial parts. In roots, the expression of AtSULTR2;1 is not affected by salinity and drought, whereas that of AtSULTR3;5 decreased significantly in response to salt stress (**Table 1**). Owing to the co-activator function of AtSULTR3;5, this may slow the allocation of sulfate to aerial parts. It is therefore possible that *Arabidopsis* adjusts the level of sulfate in roots under salt stress by modulating *AtSULTR3;5* expression. This could be part of the adaptive mechanisms used by *Arabidopsis* to load sulfate into xylem vessels while ensuring that sufficient sulfate remains in roots when uptake is limited due to high salt concentrations in soils. In *M. truncatula,* the *SULTR2;1* gene is not significantly regulated in roots in response to salt stress, but down-regulated in this tissue at early stages of water stress. The function of this transporter has not been reported yet, but if we assume a similar role to its *Arabidopsis* homolog, the down-regulation observed is likely to reflect a need to maintain sulfate in roots at these stages.

A continued loading of sulfate into xylem vessels is of paramount importance for maintaining the synthesis of sulfur molecules in aerial parts. Moreover, sulfate from the xylem acts as a chemical signal for ABA-dependent stomatal closure in leaves during early stages of water stress when ABA biosynthesis is restricted to leaves (Ernst et al., 2010). Several *SULTR* genes in **Table 1** that are regulated in shoots or leaves are good candidates for re-equilibrating the flux of sulfate in aerial parts in response to abiotic stresses. First, *SULTR2;1* is significantly down-regulated in leaves of *Arabidopsis* and *M. truncatula* subjected to drought. *AtSULTR2;1* has been shown to be not only expressed in the xylem parenchyma cells but also in the phloem cells of mature leaves, where it participates in the translocation of sulfate to young leaves (Takahashi et al., 2000). Hence, the down-regulation of *SULTR2;1* suggests a decreased flux of sulfate to young leaves, presumably to save sulfate for protection mechanisms, such as those involving ABA. Second, in *M. truncatula* subjected to drought, one *SULTR3* gene, *MtSULTR3;4*, is significantly up-regulated in aerial parts and more strongly at early stages of water stress (mild and moderate in **Table 1**). It would be of particular interest to investigate whether this transporter could play a role in leaves in controlling their early response to water stress in strong connection with ABA biosynthesis. In *Arabidopsis*, *AtSULTR3;1* and *3;4* are both significantly up-regulated in leaves subjected to salt stress, reinforcing the hypothesis raised in the previous section that both transporters could act in concert to mitigate the effect of salt stress.

Interestingly, the expression of both vacuolar *AtSULTR4* genes is significantly enhanced in leaves by drought and salinity. Moreover, *AtSULTR4;1* and *AtSULTR4;2* fall in QTL regions for tolerance to both stresses (Juenger et al., 2005; McKay et al., 2008). They are thus good candidates for multiple stress tolerance. The only *SULTR4* gene in *M. truncatula* is also up-regulated in shoots in response to drought with a statistically significant but lower fold-change compared to *Arabidopsis*. Because in *Arabidopsis*, the SULTR4 transporters were shown to enable the mobilization of the sulfate stored in the vacuoles, they may play a critical role in ensuring sulfur metabolism in plant cells when sulfate uptake is limited due to environmental constraints. Furthermore, eﬄux of sulfate from the vacuole may contribute to osmotic adjustments that play a fundamental role in water and salt stress responses. The role of SULTR4 (Kataoka et al., 2004b) has been investigated in roots but their involvement in shoots merits further investigations in relation to abiotic stress tolerance.

## REGULATION OF GENES INVOLVED IN SULFATE UPTAKE UNDER ABIOTIC STRESS CONDITIONS

The capacity of roots to take up nutrients generally declines in salt- and water-stressed plants, which may explain the changes in expression of SULTR genes belonging to groups 2, 3, and 4 under these conditions to rebalance sulfate flux between affected tissues. By examining the regulation of the two *SULTR1* genes known to control sulfate uptake in *Arabidopsis*, we observed a contrasted pattern for both genes (**Table 1**). *MtSULTR1;1* appeared down-regulated in roots subjected to both abiotic stresses, whereas *MtSULTR1;2* and *AtSULTR1;2* were up-regulated in response to salinity and drought, respectively. Barberon et al. (2008) demonstrated that *SULTR1;1* and *SULTR1;2* display unequal functional redundancy in *Arabidopsis* and left open the possibility for the *SULTR1;1* gene to display an additional function besides its role in sulfate membrane transport. Recent findings also proposed a supplementary role for AtSULTR1;2 in the regulatory or sensing/signaling pathways related to sulfur metabolism (Zhang et al., 2014b). Further studies are needed to better understand their additional function(s) and contribution to abiotic stress responses.

## AM FUNGI, A PROMISING PERSPECTIVE FOR IMPROVING SULFATE UPTAKE IN FLUCTUATING ENVIRONMENTS?

The emerging role of sulfate in plant adaptation to abiotic stresses reinforces the need to sustain proper sulfate uptake and use in cultures that face environmental stresses. One specific feature of legumes, compared to *Arabidopsis*, is their ability to perform symbiotic interactions with AM fungi. This mutualistic association is known to increase plant tolerance to drought (Augé, 2001), an abiotic stress limiting the absorption of ions, including sulfate, by roots. Recent studies in *M. truncatula* revealed that AM fungi improve sulfur nutrition in low-sulfate environments (Casieri et al., 2012; Sieh et al., 2013), probably through their capacity to take up and translocate sulfate to the root (Gray and Gerdemann, 1973; Rhodes and Gerdemann, 1978a,b; Allen and Shachar-Hill, 2009). To date, there is no information available on the regulation of plant sulfate uptake or plant sulfate transporter genes in the presence of AM fungi under drought conditions. However, because drought is associated with reduced sulfate availability, the *SULTR* genes up-regulated at low sulfate concentrations in roots colonized with AM fungi (Casieri et al., 2012; Sieh et al., 2013) might help the plant partner to survive in such environments. This is the case for *MtSULTR1;1* and *MtSULTR1;2*, both up-regulated in roots of AM symbiotic plants, especially at low sulfate concentrations (Casieri et al., 2012). Recently, Giovannetti et al. (2014) demonstrated the induction of the *LjSULTR1;2* gene during the *Lotus japonicus/Rhizophagus irregularis* mutualistic interaction, and the specific expression of this transporter in arbuscule-containing cells, strongly suggesting AM-specific sulfate transport. Investigating the regulation of such genes during AM symbiosis in response to abiotic stresses might help to decipher the roles played by these transporters in fluctuating environments.

## CONCLUSION

Several *SULTR* genes regulated by drought and/or salinity were highlighted in this review that may contribute to adjust sulfur distribution in plants subjected to abiotic stresses. We discussed their possible roles using information available in *Arabidopsis*, for which considerable advances have been made in the last two decades toward understanding SULTR functions, more recently in response to salinity (Cao et al., 2014). *SULTR* genes similarly regulated in *Arabidopsis* and *M. truncatula* are promising targets for improving sulfate transport capacities under fluctuating environmental conditions. Among these are group 3 *SULTR*, also in the list of abiotic stress-responsive genes shared between *Arabidopsis* and *M. truncatula* of Hyung et al. (2014). Group 1 SULTR are other potential targets for enhancing sulfate uptake in fluctuating environmental conditions. Members of this group were found to be up-regulated by drought stress and by AM fungi associations that increased significantly the root uptake of sulfate in low-sulfate environments, as it is the case in drought conditions. Broad collections of ecotypes and TILLING mutants are available in *M. truncatula* and in the pea crop (Dalmais et al., 2008; Le Signor et al., 2009; Deulvot et al., 2010) that can be used to study and confirm *SULTR* genes as relevant candidates for discovering favorable alleles for abiotic stress tolerance.

## Conflict of Interest Statement

The authors declare that the research was conducted in the absence of any commercial or financial relationships that could be construed as a potential conflict of interest.

## Footnotes

1 http://www.jcvi.org/medicago/

2 http://mtgea.noble.org/v3/

## References

[B1] Allen J. W. Shachar-Hill Y. (2009). Sulfur transfer through an arbuscular mycorrhiza. *Plant Physiol.* 149 549–560. 10.1104/pp.108.129866 18978070 PMC2613693

[B2] Arraouadi S. Badri M. Abdelly C. Huguet T. Aouani M. E. (2012). QTL mapping of physiological traits associated with salt tolerance in *Medicago truncatula* Recombinant Inbred Lines. *Genomics* 99 118–125. 10.1016/j.ygeno.2011.11.005 22178264

[B3] Augé R. M. (2001). Water relations, drought and vesicular-arbuscular mycorrhizal symbiosis. *Mycorrhiza* 11 3–42. 10.1007/s005720100097

[B4] Awazuhara M. Fujiwara T. Hayashi H. Watanabe-Takahashi A. Takahashi H. Saito K. (2005). The function of SULTR2;1 sulfate transporter during seed development in *Arabidopsis thaliana*. *Plant Physiol.* 125 95–105. 10.1111/j.1399-3054.2005.00543.x

[B5] Barberon M. Berthomieu P. Clairotte M. Shibagaki N. Davidian J.-C. Gosti F. (2008). Unequal functional redundancy between the two *Arabidopsis thaliana* high-affinity sulphate transporters SULTR1;1 and SULTR1;2. *New Phytol.* 180 608–619. 10.1111/j.1469-8137.2008.02604.x 18761637

[B6] Bordat A. Savois V. Nicolas M. Salse J. Chauveau A. Bourgeois M. (2011). Translational genomics in legumes allowed placing in silico 5460 unigenes on the pea functional map and identified candidate genes in *Pisum sativum* L. *G3* (Bethesda) 1 93–103. 10.1534/g3.111.000349 22384322 PMC3276132

[B7] Buchner P. Takahashi H. Hawkesford M. J. (2004). Plant sulphate transporters: co-ordination of uptake, intracellular and long-distance transport. *J. Exp. Bot.* 55 1765–1773. 10.1093/jxb/erh206 15258169

[B8] Cao M. J. Wang Z. Wirtz M. Hell R. Oliver D. J. Xiang C. B. (2013). SULTR3;1 is a chloroplast-localized sulfate transporter in *Arabidopsis thaliana*. *Plant J.* 73 607–616. 10.1111/tpj.12059 23095126

[B9] Cao M. J. Wang Z. Zhao Q. Mao J. L. Speiser A. Wirtz M. (2014). Sulfate availability affects ABA levels and germination response to ABA and salt stress in *Arabidopsis thaliana*. *Plant J.* 77 604–615. 10.1111/tpj.12407 24330104

[B10] Casieri L. Ait Lahmidi N. Doidy J. Veneault-Fourrey C. Migeon A. Bonneau L. (2013). Biotrophic transportome in mutualistic plant-fungal interactions. *Mycorrhiza* 23 597–625. 10.1007/s00572-013-0496-9 23572325

[B11] Casieri L. Gallardo K. Wipf D. (2012). Transcriptional response of *Medicago truncatula* sulphate transporters to arbuscular mycorrhizal symbiosis with and without sulphur stress. *Planta* 235 1431–1447. 10.1007/s00425-012-1645-7 22535379

[B12] Chan K. X. Wirtz M. Phua S. Y. Estavillo G. M. Pogson B. J. (2013). Balancing metabolites in drought: the sulfur assimilation conundrum. *Trends Plant Sci.* 18 18–29. 10.1016/j.tplants.2012.07.005 23040678

[B13] Dalmais M. Schmidt J. Le Signor C. Moussy F. Burstin J. Savois V. (2008). UTILLdb, a *Pisum sativum* in silico forward and reverse genetics tool. *Genome Biol.* 9 R43. 10.1186/gb-2008-9-2-r43 PMC2374714 18302733

[B14] Deulvot C. Charrel H. Marty A. Jacquin F. Donnadieu C. Lejeune-Hénaut I. (2010). Highly-multiplexed SNP genotyping for genetic mapping and germplasm diversity studies in pea. *BMC Genomics* 11:468. 10.1186/1471-2164-11-468 PMC3091664 20701750

[B15] Ding D. Zhang L. Wang H. Liu Z. Zhang Z. Zheng Y. (2009). Differential expression of miRNAs in response to salt stress in maize roots. *Ann. Bot.* 103 29–38. 10.1093/aob/mcn205 18952624 PMC2707283

[B16] El-Soda M. Kruijer W. Malosetti M. Koornneef M. Aarts M. G. (2014). Quantitative trait loci and candidate genes underlying genotype by environment interaction in the response of *Arabidopsis thaliana* to drought. *Plant Cell Environ.* 10.1111/pce.12418 [Epub ahead of print] 25074022

[B17] Ernst L. Goodger J. Q. Alvarez S. Marsh E. L. Berla B. Lockhart E. (2010). Sulphate as a xylem-borne chemical signal precedes the expression of ABA biosynthetic genes in maize roots. *J. Exp. Bot.* 61 3395–3405. 10.1093/jxb/erq160 20566566

[B18] Friesen M. L. Cordeiro M. A. Penmetsa R. V. Badri M. Huguet T. Aouani M. E. (2010). Population genomic analysis of Tunisian *Medicago truncatula* reveals candidates for local adaptation. *Plant J.* 63 623–635. 10.1111/j.1365-313X.2010.04267.x 20545888

[B19] Frugoli J. Harris J. (2001). *Medicago truncatula* on the move! *Plant Cell.* 13 458–463. 10.1105/tpc.13.3.458 11251089 PMC526011

[B20] Geng Y. Wu R. Wee C. W. Xie F. Wei X. Chan P. M. (2013). A spatio-temporal understanding of growth regulation during the salt stress response in *Arabidopsis*. *Plant Cell* 25 2132–2154. 10.1105/tpc.113.112896 23898029 PMC3723617

[B21] Giovannetti M. Tolosano M. Volpe V. Kopriva S. Bonfante P. (2014). Identification and functional characterization of a sulfate transporter induced by both sulfur starvation and mycorrhiza formation in *Lotus japonicus*. *New Phytol.* 204 609–619. 10.1111/nph.12949 25132489

[B22] Goodger J. Q. Schachtman D. P. (2010). Re-examining the role of ABA as the primary long-distance signal produced by water-stressed roots. *Plant Signal. Behav.* 5 1298–1301. 10.4161/psb.5.10.13101 20930518 PMC3115372

[B23] Gray L. E. Gerdemann J. W. (1973). Uptake of sulphur-35 by vesicular-arbuscular mycorrhizae. *Plant Soil* 39 687–689. 10.1007/BF00264184

[B24] Ha C. V. Leyva-González M. A. Osakabe Y. Tran U. T. Nishiyama R. Watanabe Y. (2014). Positive regulatory role of strigolactone in plant responses to drought and salt stress. *Proc. Natl. Acad. Sci. U.S.A.* 111 851–856. 10.1073/pnas.1322135111 24379380 PMC3896162

[B25] Huang D. Wu W. Abrams S. R. Cutler A. J. (2008). The relationship of drought-related gene expression in *Arabidopsis thaliana* to hormonal and environmental factors. *J. Exp. Bot.* 59 2991–3007. 10.1093/jxb/ern155 18552355 PMC2504347

[B26] Hyung D. Lee C. Kim J. H. Yoo D. Seo Y. S. Jeong S. C. (2014). Cross-family translational genomics of abiotic stress-responsive genes between *Arabidopsis* and *Medicago truncatula*. *PLoS ONE* 9:e91721. 10.1371/journal.pone.0091721 PMC3968010 24675968

[B27] Juenger T. E. McKay J. K. Hausmann N. Keurentjes J. J. B. Sen S. Stowe K. A. (2005). Identification and characterization of QTL underlying whole-plant physiology in *Arabidopsis thaliana*: Delta C-13, stomatal conductance and transpiration efficiency. *Plant Cell Environ.* 28 697–708. 10.1111/j.1365-3040.2004.01313.x

[B28] Kataoka T. Hayashi N. Yamaya T. Takahashi H. (2004a). Root-to-shoot transport of sulfate in *Arabidopsis*. Evidence for the role of SULTR3;5 as a component of low-affinity sulfate transport system in the root vasculature. *Plant Physiol.* 136 4198–4204. 10.1104/pp.104.045625 15531709 PMC535849

[B29] Kataoka T. Watanabe-Takahashi A. Hayashi N. Ohnishi M. Mimura T. Buchner P. (2004b). Vacuolar sulfate transporters are essential determinants controlling internal distribution of sulfate in *Arabidopsis*. *Plant Cell* 16 2693–2704. 10.1105/tpc.104.023960 15367713 PMC520965

[B30] Kawashima C. G. Matthewman C. A. Huang S. Lee B. R. Yoshimoto N. Koprivova A. (2011). Interplay of SLIM1 and miR395 in the regulation of sulfate assimilation in *Arabidopsis*. *Plant J.* 66 863–876. 10.1111/j.1365-313X.2011.04547.x 21401744

[B31] Kilian J. Whitehead D. Horak J. Wanke D. Weinl S. Batistic O. (2007). The AtGenExpress global stress expression data set: protocols, evaluation and model data analysis of UV-B light, drought and cold stress responses. *Plant J.* 50 347–363. 10.1111/j.1365-313X.2007.03052.x 17376166

[B32] Krusell L. Krause K. Ott T. Desbrosses G. Krämer U. Sato S. (2005). The sulfate transporter SST1 is crucial for symbiotic nitrogen fixation in Lotus japonicus root nodules. *Plant Cell* 17 1625–1636. 10.1105/tpc.104.030106 15805486 PMC1091779

[B33] Kumar S. Asif M. H. Chakrabarty D. Tripathi R. D. Trivedi P. K. (2011). Differential expression and alternative splicing of rice sulphate transporter family members regulate sulphur status during plant growth, development and stress conditions. *Funct Integr Genomics* 11 259–273. 10.1007/s10142-010-0207-y 21221698

[B34] Le Signor C. Savois V. Aubert G. Verdier J. Nicolas M. Pagny G. (2009). Optimizing TILLING populations for reverse genetics in *Medicago truncatula*. *Plant Biotechnol. J.* 7 430–441. 10.1111/j.1467-7652.2009.00410.x 19490506

[B35] Li D. Su Z. Dong J. Wang T. (2009). An expression database for roots of the model legume *Medicago truncatula* under salt stress. *BMC Genomics* 10:517. 10.1186/1471-2164-10-517 PMC2779821 19906315

[B36] McKay J. K. Richards J. H. Nemali K. S. Sen S. Mitchell-Olds T. Boles S. (2008). Genetics of drought adaptation in *Arabidopsis thaliana* II. QTL analysis of a new mapping population, Kas-1 × Tsu-1. *Evolution* 62 3014–3026. 10.1111/j.1558-5646.2008.00474.x 18691264

[B37] Nishiyama R. Le D. T. Watanabe Y. Matsui A. Tanaka M. Seki M. (2012). Transcriptome analyses of a salt-tolerant cytokinin-deficient mutant reveal differential regulation of salt stress response by cytokinin deficiency. *PLoS ONE* 7:e32124. 10.1371/journal.pone.0032124 PMC3280229 22355415

[B38] Pandey N. Ranjan A. Pant P. Tripathi R. K. Ateek F. Pandey H. P. (2013). CAMTA 1 regulates drought responses in *Arabidopsis thaliana*. *BMC Genomics* 14:216. 10.1186/1471-2164-14-216 PMC3621073 23547968

[B39] Perera I. Y. Hung C. Y. Moore C. D. Stevenson-Paulik J. Boss W. F. (2008). Transgenic *Arabidopsis* plants expressing the type 1 inositol 5-phosphatase exhibit increased drought tolerance and altered abscisic acid signaling. *Plant Cell* 20 2876–2893. 10.1105/tpc.108.061374 18849493 PMC2590728

[B40] Rhodes L. H. Gerdemann J. W. (1978a). Hyphal translocation and uptake of sulfur by vesicular-arbuscular mycorrhizae of onion. *Soil Biol. Biochem.* 10 355–360. 10.1016/0038-0717(78)90057-3

[B41] Rhodes L. H. Gerdemann J. W. (1978b). Influence of phosphorus nutrition on sulphur uptake by vesicular arbuscular. *Soil Biol. Biochem.* 10 361–364. 10.1016/0038-0717(78)90058-5

[B42] Roux B. Rodde N. Jardinaud M. F. Timmers T. Sauviac L. Cottret L. (2014). An integrated analysis of plant and bacterial gene expression in symbiotic root nodules using laser-capture microdissection coupled to RNA sequencing. *Plant J.* 77 817–837. 10.1111/tpj.12442 24483147

[B43] Sieh D. Watanabe M. Devers E. A. Brueckner F. Hoefgen R. Krajinski F. (2013). The arbuscular mycorrhizal symbiosis influences sulfur starvation responses of *Medicago truncatula*. *New Phytol.* 197 606–616. 10.1111/nph.12034 23190168

[B44] Takahashi H. Watanabe-Takahashi A. Smith F. W. Blake-Kalff M. Hawkesford M. J. Saito K. (2000). The roles of three functional sulphate transporters involved in uptake and translocation of sulphate in *Arabidopsis thaliana*. *Plant J.* 23 171–182. 10.1046/j.1365-313x.2000.00768.x 10929111

[B45] Varin S. Cliquet J. B. Personeni E. Avice J.-C. Lemauviel-Lavenant S. (2010). How does sulphur availability modify N acquisition of white clover (Trifolium repens L.)? *J. Exp. Bot.* 61 225–234. 10.1093/jxb/erp303 19933318 PMC2791126

[B46] Wang Y. Yang L. Zheng Z. Grumet R. Loescher W. Zhu J. K. (2013). Transcriptomic and physiological variations of three *Arabidopsis* ecotypes in response to salt stress. *PLoS ONE* 8:e69036. 10.1371/journal.pone.0069036 PMC3720874 23894403

[B47] Wilkinson S. Davies W. J. (2002). ABA-based chemical signaling: the co-ordination of responses to stress in plants. *Plant Cell Environ.* 25 195–210. 10.1046/j.0016-8025.2001.00824.x 11841663

[B48] Xiong L. Ishitani M. Lee H. Zhu J. K. (2001). The *Arabidopsis* LOS5/ABA3 locus encodes a molybdenum cofactor sulfurase and modulates cold stress- and osmotic stress-responsive gene expression. *Plant Cell* 13 2063–2083. 10.1105/tpc.13.9.2063 11549764 PMC139452

[B49] Yoshimoto N. Inoue E. Saito K. Yamaya T. Takahashi H. (2003). Phloem-localizing sulfate transporter, Sultr1;3, mediates re-distribution of sulfur from source to sink organs in *Arabidopsis*. *Plant Physiol.* 131 1511–1517. 10.1104/pp.014712 12692311 PMC166910

[B50] Yoshimoto N. Inoue E. Watanabe-Takahashi A. Saito K. Takahashi H. (2007). Posttranscriptional regulation of high-affinity sulfate transporters in *Arabidopsis* by sulfur nutrition. *Plant Physiol.* 145 378–388. 10.1104/pp.107.105742 17720755 PMC2048716

[B51] Zhang J. Y. Cruz D. E. Carvalho M. H. Torres-Jerez I. Kang Y. Allen S. N. (2014a). Global reprogramming of transcription and metabolism in *Medicago truncatula* during progressive drought and after rewatering. *Plant Cell Environ.* In press (free access online). 10.1111/pce.12328 [Epub ahead of print] PMC4260174 24661137

[B52] Zhang B. Pasini R. Dan H. Joshi N. Zhao Y. Leustek T. (2014b). Aberrant gene expression in the *Arabidopsis* SULTR1;2 mutants suggests a possible regulatory role for this sulfate transporter in response to sulfur nutrient status. *Plant J.* 77 185–197. 10.1111/tpj.12376 24308460

[B53] Zhang X. Lu G. Long W. Zou X. Li F. Nishio T. (2014c). Recent progress in drought and salt tolerance studies in *Brassica* crops. *Breed Sci.* 64 60–73. 10.1270/jsbbs.64.60 24987291 PMC4031111

[B54] Zhou L. Liu Y. Liu Z. Kong D. Duan M. Luo L. (2010). Genome-wide identification and analysis of drought-responsive microRNAs in Oryza sativa. *J. Exp. Bot.* 61 4157–4168. 10.1093/jxb/erq237 20729483

[B55] Zuber H. Davidian J.-C. Aubert G. Aimé D. Belghazi M. Lugan R. (2010). The seed composition of *Arabidopsis* mutants for the group 3 sulfate transporters indicates a role in sulfate translocation within developing seeds. *Plant Physiol.* 154 913–926. 10.1104/pp.110.162123 20702726 PMC2949013

